# Higher Antipsychotic Usage Among Sexual Minorities with Cognitive Impairment: Evidence from the “All of Us” Study

**DOI:** 10.1093/geroni/igaf122.2034

**Published:** 2025-12-31

**Authors:** Addam Reynolds

**Affiliations:** University of Southern California Leonard Davis School of Gerontology, Los Angeles, California, United States

## Abstract

Antipsychotics are often used to treat the behavioral and psychotic symptoms of Alzheimer’s disease and related dementia (ADRD); however, such use comes with increased risk for adverse events, such as death and cognitive impairment. Absent from this literature are considerations that sexual minorities are more likely to have a treatment history with antipsychotics due to comorbid mental health diagnoses. The current study addresses this gap in the literature by testing if sexual minorities are more likely than their heterosexual counterparts to have a treatment history of antipsychotic usage. This study leverages data from the All of Us Research Program. We restricted our sample to those 65 and over, and those with a clinical finding of impaired cognition, or a diagnosis of mild cognitive impairment or ADRD listed at least twice in participant’s electronic health records (N = 9,358). We use linear regression to test the study’s hypothesis. Sexual minorities with cognitive impairment, in comparison to heterosexuals, have 31.2% (95% CI: 1.063 – 1.6120; p < .05) higher odds of having a treatment history of antipsychotics, even after controlling for demographic information (e.g. race/ethnicity, age, sex assigned at birth, and education) and co-morbid mental health diagnoses. Sexual minorities with cognitive impairment are more likely to have a treatment history of antipsychotics, in comparison to their heterosexual counterparts. Future research is needed to determine why sexual minorities with cognitive impairment are more likely to be prescribed drug class that places them at higher risk for adverse events.

